# Long‐term spatio‐temporal changes in a West African bushmeat trade system

**DOI:** 10.1111/cobi.12545

**Published:** 2015-06-23

**Authors:** J. McNamara, J. M. Kusimi, J. M. Rowcliffe, G. Cowlishaw, A. Brenyah, E. J. Milner‐Gulland

**Affiliations:** ^1^ Imperial College London, Division of BiologySilwood Park Campus, Manor HouseBuckhurst Road, AscotBerksSL5 7PYUnited Kingdom; ^2^ Institute of ZoologyZoological Society of LondonRegents ParkLondonNW1 4RYUnited Kingdom; ^3^ University of GhanaLegonAccraGhana; ^4^ Ghana Wildlife DivisionForestry CommissionAccraGhana

**Keywords:** Africa, conservation planning, ecosystem management, forest, land‐cover change, land‐use change, land‐use planning, remote sensing, África, bosque, cambios en la cobertura de suelo, cambios en el uso de suelo, detección remota, manejo de ecosistemas, planeación de la conservación, planeación del uso de suelo

## Abstract

Landscapes in many developing countries consist of a heterogeneous matrix of mixed agriculture and forest. Many of the generalist species in this matrix are increasingly traded in the bushmeat markets of West and Central Africa. However, to date there has been little quantification of how the spatial configuration of the landscape influences the urban bushmeat trade over time. As anthropogenic landscapes become the face of rural West Africa, understanding the dynamics of these systems has important implications for conservation and landscape management. The bushmeat production of an area is likely to be defined by landscape characteristics such as habitat disturbance, hunting pressure, level of protection, and distance to market. We explored (SSG, tense) the role of these four characteristics in the spatio‐temporal dynamics of the commercial bushmeat trade around the city of Kumasi, Ghana, over 27 years (1978 to 2004). We used geographic information system methods to generate maps delineating the spatial characteristics of the landscapes. These data were combined with spatially explicit market data collected in the main fresh bushmeat market in Kumasi to explore the relationship between trade volume (measured in terms of number of carcasses) and landscape characteristics. Over time, rodents, specifically cane rats (Thryonomys swinderianus), became more abundant in the trade relative to ungulates and the catchment area of the bushmeat market expanded. Areas of intermediate disturbance supplied more bushmeat, but protected areas had no effect. Heavily hunted areas showed significant declines in bushmeat supply over time. Our results highlight the role that low intensity, heterogeneous agricultural landscapes can play in providing ecosystem services, such as bushmeat, and therefore the importance of incorporating bushmeat into ecosystem service mapping exercises. Our results also indicate that even where high bushmeat production is possible, current harvest levels may cause wildlife depletion.

## Abstract (pt)

Cambios Espacio‐Temporales a Largo Plazo en un Sistema de Mercado de Carne de Animales Silvestres en África Occidental

**Resumen**

Los paisajes en muchos de los países en desarrollo consisten de una matriz heterogénea de agricultura mixta y bosque. Muchas de las especies generalistas en esta matriz cada vez son más comercializadas en los mercados de carne de animales silvestres de África central y occidental. Sin embargo, a la fecha ha habido poca cuantificación de cómo influye la configuración espacial del paisaje al mercado urbano de carne de animales silvestres con el tiempo. Conforme los paisajes antropogénicos se vuelven comunes en el África occidental rural, entender las dinámicas de estos sistemas tiene implicaciones importantes para la conservación y el manejo del paisaje. Es posible que la producción de esta carne en un área esté definida por características del paisaje como la perturbación del hábitat, la presión de caza, el nivel de protección y la distancia al mercado. Exploramos el papel de estas cuatro características en las dinámicas espacio‐temporales del mercado comercial de la carne de animales salvajes cerca de la ciudad de Kumasi, Ghana, durante 27 años (1978 a 2004). Usamos métodos de sistemas de información geográfica (SIG) para generar mapas que delinearan las características espaciales de los paisajes. Estos datos se combinaron con datos de mercado espacialmente explícitos colectados en el principal mercado de carne fresca de animales salvajes en Kumasi para explorar la relación entre el volumen comercial (medido en términos de número de cadáveres) y las características del paisaje. Con el tiempo, los roedores, específicamente la rata de la caña (Thryonomys swinderianus) se volvieron más abundantes en el mercado con relación a los ungulados y la zona de influencia del mercado se expandió. Las áreas de perturbación intermedia suministraron más carne de animales silvestres, pero las áreas protegidas no tuvieron efecto. Con el tiempo, las zonas con un alto nivel de cacería mostraron declinaciones significativas en el suministro de carne de animales silvestres. Nuestros resultados resaltan el papel que pueden tener los paisajes de agricultura heterogénea y de baja intensidad en la aportación de servicios ambientales, como este tipo de carne, y por lo tanto la importancia de incorporar la carne de animales salvajes a los ejercicios de mapeo de servicios ambientales. Nuestros resultados también indican que incluso donde una producción alta de este tipo de carne es posible, los niveles actuales de recolección pueden causar una reducción en la vida silvestre.

## Introduction

Anthropogenic landscapes, and the ecosystem services and benefits they provide, are dynamic in both time and space and driven by factors such as human demography, urbanization, climate, and land‐use. Understanding these dynamics is important for designing effective conservation and land management strategies that take account of the variety of benefits humans derive from such landscapes and the trade‐offs associated with different management strategies (Anderson et al. 2009; Armsworth et al. 2012). Bushmeat is an important benefit provided by ecosystems in the tropics. For example, in Ghana, the commercial trade has been estimated to be worth over US$350 million dollars per annum (Ntiamoa‐Baidu 1998). Yet to date, no studies have explored how changes in the spatial characteristics of the landscape influence commercial bushmeat production over time. Consequently bushmeat is rarely incorporated into ecosystem services evaluations or land management strategies (Holbech 2001). Assessing such dynamics is key if the true benefits derived by people from the land are to be incorporated into landscape‐level conservation planning exercises.

We explored how the spatio‐temporal dynamics of an anthropogenic tropical forest landscape in Ghana have influenced the commercial bushmeat trade in the region. We identified 4 characteristics of the landscape that are likely to influence the dynamics of the bushmeat trade: habitat disturbance, level of protection, hunting pressure, and distance to market. First, disturbed tropical forest landscapes can, under certain circumstances, be more productive for bushmeat or game production than undisturbed climax vegetation (Robinson & Bennett 2004). Opening the canopy can improve browsing conditions for lower story herbivores, particularly rodents and ungulates, whereas forest mosaics interspersed with food crops and mixed agricultural landscapes can harbor a high concentration of edible foods of particular benefit to more disturbance‐resilient generalists (Jorgenson 2000).

Second, unsustainable hunting pressure can reduce wildlife populations below the level of minimum viability and alter the species composition of wildlife communities such that larger, less fecund species are extirpated (Robinson et al. 1999; Naughton‐Treves et al. 2003; Albrechtsen et al. 2007). Over time, locations that are susceptible to extreme levels of exploitation are likely to experience a decline in trade volumes and shifts in species composition relative to less heavily hunted locations (e.g., Robinson et al. 1999; Fitzgibbon et al. 2000; Jorgenson 2000). The most obvious shifts in species composition may be an increase in the relative number of prolific and disturbance‐resilient rodents that can withstand high hunting pressure. This shift can be measured through the rodent:ungulate ratio (Wilkie & Carpenter 1999; Rowcliffe et al. 2003). If hunting pressure is unsustainable, an increase in the rodent:ungulate ratio can be expected across all hunting areas.

Third, protected areas may also influence the spatial patterns of exploitation by human hunters. Bushmeat harvest per inhabitant and distance from protected areas are inversely related across urban and rural markets in Nigeria and Cameroon, particularly for species more vulnerable to harvesting, such as forest‐dependent primates and large ungulates (Fa et al. 2006).

Fourth, a location's distance from market also plays an important role in defining both the trade volume and species composition originating from that location. For example, across Africa hunters who are farther from urban centers sell less of their bushmeat (Brashares et al. 2011). In Equatorial Guinea, distance from markets affects which species are brought to market (Allebone‐Webb et al. 2011). Trade with hunters in isolated locations maximized trader profits, whereas trade with hunters close to markets maximized hunter profits. In addition, over time, the size of a market's catchment area may change, as either local resources are depleted or new actors enter the trade (Clayton et al. 1997; Crookes et al. 2005). On Bioko Island, Equatorial Guinea, increases in catchment size are associated with faunal depletion (Albrechtsen et al. 2007).

If appropriate land management policies are to be developed that take into account trade‐offs between conflicting land‐use objectives, it is imperative that the consequences of changing patterns of land‐use for wildlife, and for human use of wildlife, be understood. Using the bushmeat trade in the city of Kumasi, Ghana, as a case study, we tested hypotheses about the role of these 4 landscape characteristics in the spatio‐temporal dynamics of the commercial bushmeat trade over 27 years (Table 1). The landscape around Kumasi was subject to intense conversion over the study period and is now primarily defined by agriculture, with much of the remaining tropical forest confined to forest reserves and wildlife protected areas (Braimoh 2009). In addition, a long‐running study of the Atwemonom bushmeat market in Kumasi between 1978 and 2004 means that long‐term spatially explicit data are available on the bushmeat trade.

**Table 1:** Spatio‐temporal characteristics of the bushmeat trade and their associated hypotheses and predictions

Spatial		Hypotheses (numbers) and	
characteristic	Summary description	predictions (letters)	References
Habitat disturbance	Harvest rates and biological production are expected to vary with changes in human‐induced disturbance in the landscape.	1. Bushmeat off‐take will be greatest in semidisturbed landscapes. a. Bushmeat volumes will be quadratically related to level of disturbance. b. Trade volumes of generalist species, such as rodents, will be less sensitive to higher levels of disturbance than other species groups.	Robinson & Bennett 2004
Hunting pressure	High levels of hunting pressure (proxied by source overlap) may reduce standing wildlife biomass and alter species composition toward smaller bodied mammals.	2. Heavily hunted areas will experience reduced harvest rates and altered species composition. a. Trade volumes from areas with a high density of hunting settlements will decline over time. b. The ratio of rodents to ungulates will be greater in areas with high source overlap, and this effect will increase over time. c. The rodent:ungulate ratio will increase over time across all areas.	Rowcliffe et al. 2003; Naughton‐Treves et al. 2003; Jorgenson 2000
Protected areas	Protected areas (PAs) may act as refuges for wildlife and thus be associated with both illegal hunting within their boundaries and spillover effects (whereby hunters benefit from wildlife emigrating from the reserve into surrounding areas) outside them. Both processes may lead to higher off‐takes for vulnerable species (such as ungulates) but not for more generalist species (such as rodents).	3a. Bushmeat off‐take of certain species will be higher in communities close to protected areas—Protected area presence will be positively correlated with ungulate trade volumes but not correlated with rodent trade volumes.	Fa et al. 2006
Distance to market	Distance to market represents a potential barrier to participating in the commercial trade. This may influence both the species that are brought to market and the degree to which otherwise productive landscapes participate in the trade. Over time, as resources become depleted and urban demand grows, one would expect incentives to exploit more distant resources to increase.	4. The spatial pattern of market supply will change over time. a. Distance to market will be negatively correlated to trade volumes. b. Catchment area of the market will increase over time.	Crookes et al. 2005; Allebone‐Webb et al. 2011; Albrechtsen et al. 2007; Brashares et al. 2011

## Methods

Kumasi, Ghana's second largest city (population 2 million), is located in the Ashanti Region. Fresh and smoked bushmeat is sold from various locations across the city. We focused on the main bushmeat market at Atwemonom that deals solely in fresh meat. The decision to use Atwemonom as a case study was based on the availability of 26 years of market data. Atwemonon is renowned as one of largest fresh bushmeat markets in West Africa (Falconer 1992) and has been the subject of several previous studies (Falconer 1992; Ntiamoa‐Baidu 1998; Hofmann et al. 1999; Crookes et al. 2005). Additionally, the Atwemonom market and surrounding hunting communities were surveyed on three occasions by J.M. and A.B. between 2010 and 2012 (McNamara 2014).

### Bushmeat Market Data

Between May 1978 and June 2004, Ghana Wildlife Division (GWD) staff regularly visited the Atwemonom market to record details of the trade in fresh bushmeat. On days when the market was monitored, GWD personnel were present from opening until closing and directly recorded information on species, method of kill, and the village of origin of the hunter. Data were collected through interviews of hunters or traders, and the aim was to cover all animals brought to the market (Supporting Information). These data were used to identify the volume (number of carcasses), species, and location of capture (the geo‐referenced source location) of bushmeat sold in the market. The geo‐referenced data set consisted of 46,769 records covering 2,527 days recorded over 27 years from 1978 to 2004. All data relate to the open season, which runs for 8 months from December to July, during which hunting is permitted for all species, except those classified in schedule 1 (Wildlife Conservation Regulations 1971). Data from the closed season, during which hunting is prohibited for all species except the cane rat (*Thryonomys swinderianus*) were excluded due to concerns over reliability because there is likely to be misreporting of trade in prohibited species during this time (Crookes et al. 2005; A.B., personal observation).

### Model Data Selection

Ideally, patterns of covariation between the bushmeat trade and relevant landscape characteristic predictors would have been examined across all years. However, land cover and therefore habitat disturbance (hypothesis 1) could be quantified only for 1986 and 2002, when sufficiently high‐quality (cloud free) satellite images were available. Consequently, we focused primarily on these 2 years. There were 2 exceptions: the analyses of changes in catchment area (prediction 4b, Table 1) and species composition (prediction 3c), for which the full data set could be used. Consequently, 2 spatially explicit databases were produced: one relating to all market records relevant to the years 1986 and 2002 and the second relating to records from all years. Two hunting seasons were included for both 1986 and 2002 to maximize the accuracy of the bushmeat market records for those years (Table 2). A summary of species traded during this period is in Supporting Information.

**Table 2:** Summary statistics of bushmeat market records from Atwemonom Market, Kumasi, Ghana

	Land use		
Data	1986	2002	Catchment (all years)
Period covered	Dec 85–Jul 86 & Dec 86–Jul 87	Dec 01–Jul 02 & Dec 02–Jul 03	Dec–Jul, 1978–2004
Total records*	4,647	2,875	46,769
Geo‐referenced	4,437	2,771	43,550
Records geo‐referenced (%)	95.5	96.4	93.1
Unique source locations	203	167	389
Mean number of carcasses per source per day (CV%)	23.4	17.3	11.7
	(157)	(161)	(189)
Median number of carcasses per source per day	8	5	5

*Each record is of a single carcass.

### Defining and Describing Source Locations

Settlements serving the market (source locations) were geographically located in a two‐part process. First, the identification of source locations was reviewed for spelling errors in consultation with the Ghana Wildlife Division (GWD) staff member responsible for their original collection (AB). Second, geographical coordinates were assigned using a database of village locations in the region compiled by the Ghana Survey Department.

To define the 4 landscape characteristics tested for each source location, we identified the hunting catchment (hereafter radius) of the source location and the landscape characteristics in that radius. We imported the bushmeat data, summarized according to source location, into ArcGIS and projected in UTM WGS 1984. A 7‐km‐radius hunting zone was produced around each source to represent the hunting radius. We chose 7 km based on interviews with 53 hunters in 2011 in 2 communities near Kumasi, where the average distance traveled to the outer extent of their hunting grounds was 7.7 km (SE 4.8) (Alexander et al. 2014). Source locations with <3 market records were excluded because low numbers of records could be due more to irregular observer effort than to landscape characteristics. For example, in 1986 observers were present in the market on approximately 71% of days, whereas in 2002 observers were present on 40% of days (Supporting Information). Because there was a substantial number of sites with few records (20% and 26% of communities had <3 records in 1986 and 2002 respectively) there was a risk that the large number of minimally sampled sites distributed across the landscape would obscure the fundamental patterns. The cut‐off value was low due to the data being heavily skewed toward small bushmeat volumes (Table 2). Sources within 2 km of each other were merged into a single source to minimize discrepancies where two or more neighboring sources shared more than 90% of the same hunting area (defined by the 7‐km radius) but produced substantially different bushmeat volumes. The difference in volumes between villages in these cases was more likely to reflect local livelihood choices or trader habits (e.g., visiting one village to collect bushmeat from all nearby villages) than variation in the landscape (A.B., personal observation).

### Measuring Habitat Disturbance

Prior to measuring habitat disturbance for each source (in the hunting radius), the sources were assigned to either the tropical forest or savannah zone with a geo‐referenced map of Ghana's ecological zones supplied by the geographic information system (GIS) unit of the Resource Management Support Centre of the Ghana Forestry Commission. Two sources supplied Kumasi from the northeastern savannah and were excluded from analyses because the hypothesized quadratic relationship between bushmeat production and habitat disturbance applies only in tropical forests (Robinson & Bennett 2004). For the remaining sources, located in the tropical forest zone, habitat disturbance was assessed through land‐cover change quantified from satellite images.

Semiprocessed (to correct for atmospheric differences between images), geo‐referenced Landsat satellite images were obtained online from the U.S. Geological Survey Global Visualization Viewer (Glovis) for 1986 and 2002. Data for 1986 were from the Landsat Multispectral Scanner (MSS) and for 2002 were from the Landsat Thematic Mapper scanner (TM). The Kumasi catchment area, as defined by the bushmeat market data, covered an area of 39,204 km^2^ (198 × 198 km) and intersected 4 Landsat scenes. Images were prepared in IDRISI (for details see Supporting Information). Supervised classification techniques were used, based on a ground truthing exercise conducted in one part of the study area. Land‐cover information collected during the ground‐truthing exercise was augmented with data from Google Earth and consultation with experts familiar with the region at the Department of Geography, University of Ghana.

Six land classes were defined: closed canopy forest, open canopy forest and tree crops, farmland, savannah woodland (being derived in the forest zone due to landscape degradation and natural in savannah zone), settlements and bare earth, and water. Cloud, which was present in a small section of one scene in 2002 (representing <6.4% of the image area), was classified as no data. The separability of the 6 digitized classes was quantified and confirmed as satisfactory for all classes with the Jeffries–Matusita distance (1.8 < J‐M < 1.98). Scenes were classified using a maximum‐likelihood modeling routine and subsequently composited into a single unified image (Supporting Information).

The land‐cover classification highlighted significant growth in the coverage of derived savannah woodland between 1986 and 2002 across the region as a whole. Although sources within the savannah zone itself were excluded, derived savannah woodland reflected forest degradation and was therefore included in the analyses. Each land class was ranked in order of disturbance: 1, closed canopy forest, relatively undisturbed; 2, open canopy forest and tree crops together with derived savannah woodland, low‐to‐moderate disturbance; 3, farm and fallow lands, moderate‐to‐high disturbance; and 4, settlements and bare earth, highly disturbed. The mean disturbance index for each source, *E*(*D*), was calculated as

(1) $$E\left(D\right)=\left[\sum_{i=1}^{n}d_{i}.P_{i}\right]/n,$$

where $d_{i}$ is the discrete disturbance index of land class *i*, $P_{i}$ is the proportion of land class *i* in the source hunting radius ($\sum_{i=1}^{n}P_{i}\;=1$), and *n* is a normalizing constant equal to the number of discrete indices (4 in this instance).

### Measuring Hunting Pressure

In the absence of direct information on hunting pressure, we used the density of source locations as a proxy on the basis that the number of hunters per location would be relatively homogeneous over the landscape, which we call source overlap. Thus, areas with a higher source overlap were likely to experience higher hunting pressure. This assumption is based on previous studies in the region (Falconer 1992; Crookes et al. 2007) and personal observation. The density of source locations was described in terms of an index that represented the overlap between neighboring sources hunting radii. Thus, an isolated village, with a 7‐km hunting radius overlapped with no other source, would have a source overlap of 1 km^2^. The maximum source overlap was 6.8 km^2^ and the median was 3.2 km^2^.

### Measuring Protected Area Coverage and Distance to Market

To calculate measures of protected area coverage and distance to market, two additional map layers were produced. First, a digital map of national parks and forest reserves, supplied by the Ghana Survey Department, was used to define the boundaries of the protected areas in the region in ArcGIS. Access to protected areas was described in terms of the proportion of protected land within a given source's hunting radius. Protected areas in this landscape largely comprise forest reserves utilized for timber extraction. Of 98 reserves, 5 were fully protected and designated as strict nature reserves—Kogyae Strict Nature Reserve, Boabeng‐Fiema Monkey Sanctuary, Bobiri Wildlife Sanctuary, Bomfobiri Wildlife Sanctuary, and Owabi Wildlife Sanctuary. Second, a map of the local road network was obtained from CERSGIS, University of Ghana. Distance to market was extracted using a network analysis in ArcGIS to measure the shortest route along the local road network between source and market. The quality of roads was likely to vary across space and time, but information on road quality was not available; therefore, all roads were treated equally.

### Statistical Analyses

We tested the predictions of our 4 hypotheses (Table 1) with general linear models (to account for the non‐normal nature of the data, owing to the data being skewed toward smaller records [Table 2]), a quasi‐poisson error structure with log link for over‐dispersed count data, and an offset to account for differences in observer effort between years (number of days the market was observed in each period). Four response variables were derived for each source location: trade volume (total number of carcasses) of all species, rodent species, and ungulate species and the rodent:ungulate ratio. Our predictor variables were habitat disturbance (hypothesis 1 [Table 1]), source overlap (hypothesis 2), protected area coverage (hypothesis 3), and distance to market (hypothesis 4) at each source location. Year was also included as a predictor, as were interactions hypothesized a priori as potentially important (Supporting Information).

Potential collinearity between the predictor variables was explored in a correlation matrix (Supporting Information). A strong correlation (0.79) was present in the relationship between distance to market and source overlap. As might be expected, settlements were denser closer to Kumasi. Despite concerns of collinearity, however, both variables were retained due to the specific hypotheses associated with each. Interpretation of the results is made with awareness of the potentially confounding issue of collinearity between these variables.

The general formula for the model describing bushmeat supply (*V*) at location *j*, excluding interaction terms, took the form

(2) $$\begin{array}{ccc} V_{j}\; & ∼ & \;d_{j}+d_{j}^{2}+h_{j}+r_{j}+l_{j}+y_{j}+\mathrm{offset}\;\left(\left[\log\;(e_{j})\right]\right)\;\;\;\\ & & j=1,....\;,J, \end{array}$$

where trade volumes are described in terms of a quadratic relationship with disturbance$\;(d_{j})$, linear relationships with source overlap $\left(h_{j}\right)$, protected area coverage $\left(r_{j}\right)$, distance to market $\left(l_{j}\right)$, year $\left(y_{j}\right)$, and observer effort $\left(e_{j}\right)$.

Interactions were selectively included based on our hypotheses (Table 1) and knowledge of the system. Two‐way interactions were included between source overlap and year (hypothesis 2); protected area and year, protected area and distance, and protected area and source overlap (hypothesis 3); and distance and year (hypothesis 4). Models were tested and simplified through the removal of nonsignificant independent variables (later reintroduced after the selection of the optimal model to verify there was no effect). Minimum adequate models were selected by way of a stepwise model selection based on *F* tests.

We used univariate statistics (R, Stats package) to examine how the rodent:ungulate ratio (prediction 2c [Table 1]) and the spatial characteristics of the catchment area (prediction 4b) changed over time (1978 to 2004) for the full range of the data.

## Results

**Figure 1:**
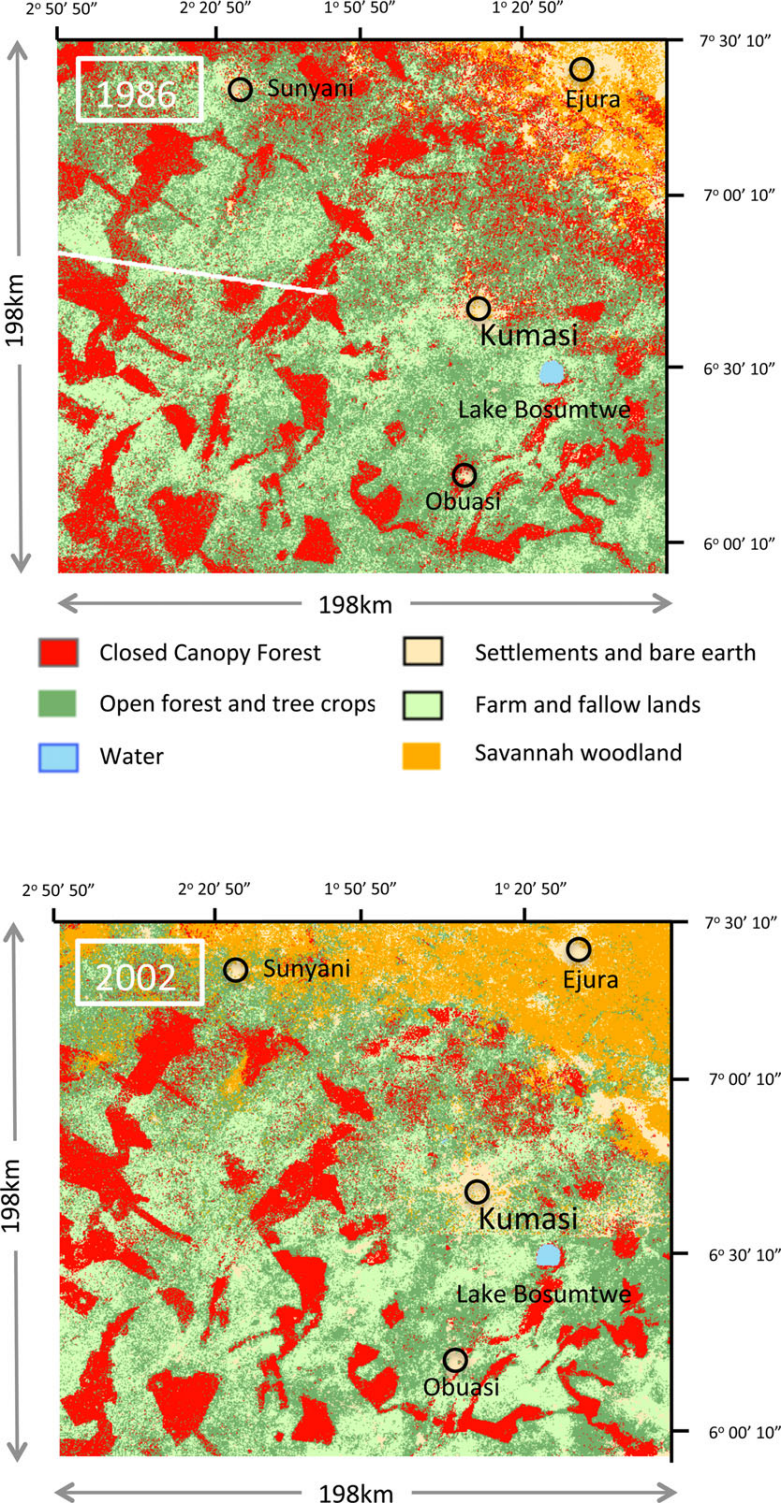
Maps of the catchment area associated with the Atwemonom bushmeat market, Kumasi, in 1986 and 2002, classified according to different land‐use types.

Changes in land cover within the catchment area between 1986 and 2002 indicated increased levels of human activity and disturbance. The area of closed canopy forest and open canopy forest declined by 7% and 6% respectively (Fig. 1). Most dramatically, the area of closed canopy forest outside of reserves declined by 47%. Conversely there was an increase in land attributed to settlements and bare earth (3%) and savannah (largely derived savannah in the forest zone, 7%). There was a marginal increase in the area of farm and fallow lands (1%). In 2002, 71% of closed canopy forest was contained in 98 protected areas with an average area of 76 km^2^ (range 1.75– 414 km^2^). Of these protected areas, 7 (1.5% of the total catchment area) were not managed for timber production. The rural landscape of the catchment area around Kumasi is therefore strongly defined by human disturbance, with only a small fraction of the landscape protected from commercial extractive activities.

**Figure 2:**
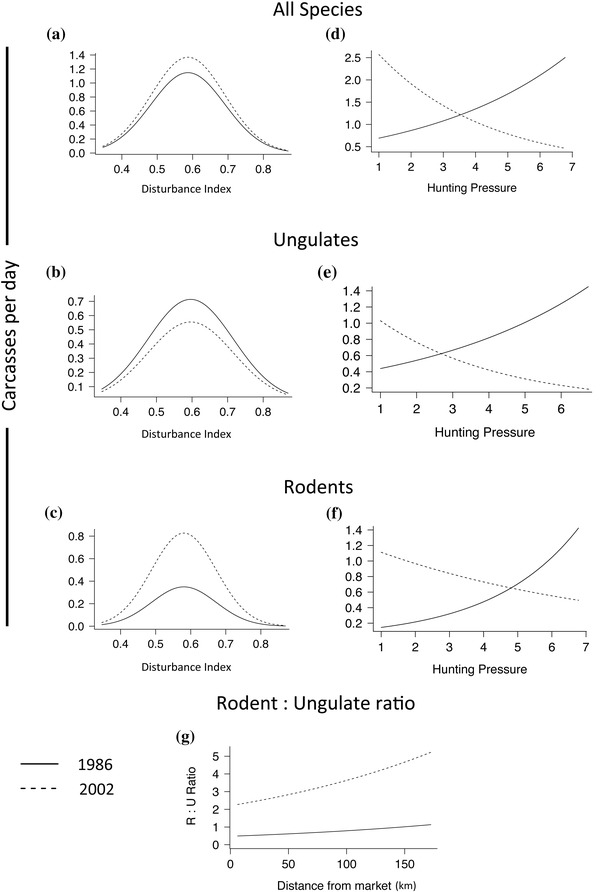
Fitted relationships describing variation in trade volumes of all, ungulate, and rodent species by year characterized by (a–c) habitat disturbance and (d–f) hunting pressure (1, lowest pressure; 7, highest pressure). (g) Variation in the rodent (R):ungulate (U) ratio by year and characterized according to distance from market. Values plotted are predictions of the generalized linear models.

**Figure 3:**
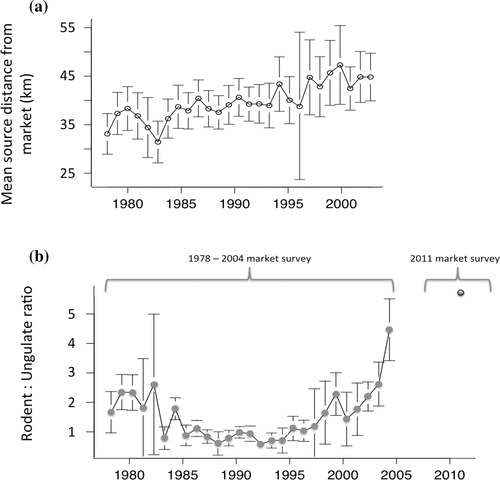
(a) Mean distance from Kumasi market of communities supplying bushmeat (error bars, 95% CI) and (b) variation in the ratio of rodents to ungulates over time in Kumasi market. Data are drawn from both the 1978–2004 Atwemonom market survey and an independent survey of the market conducted by the authors in 2011 (Supporting Information). Due to a small sample in 1978, the first year shown is 1979.

Trade in all species groups peaked in semidisturbed landscapes (Figs. 2a–c), in support of the habitat disturbance hypothesis, prediction 1a (Table 1). There was no evidence that rodent trade volumes were less sensitive to high levels of disturbance, contrary to prediction 1b (Figs. 2b & 2c). In 1986 trade volumes of all species increased with source overlap (Figs. 2d–f). However, this relationship reversed in 2002, when trade volumes were negatively correlated with source overlap, in support of the hunting pressure hypothesis, prediction 2a. Rodent trade volumes were more stable at high levels of source overlap than ungulate trade volumes, as indicated by the intersection between the 1986 and 2002 trade volume relationships (Figs. 2e & 2f). However, the rodent:ungulate ratio was independent of source overlap, contrary to prediction 2b. The rodent:ungulate ratio increased between 1986 and 2002, which is consistent with prediction 2c (Fig. 2g, Table 3). This observation is supported by the fact that although total and rodent trade volumes increased between 1986 and 2002, ungulate trade volumes declined (Figs. 2a–c). An increase in the rodent:ungulate ratio also occurred over the full survey period (Fig. 3b), from 2.3 in 1979 to 4.7 in 2004 (*t* = –2.53, df = 314, *p* = 0.01). There was no relationship between the presence of reserves and trade volumes for any species group, contrary to the protected areas hypothesis, prediction 3a (Table 3). Total and ungulate trade volumes declined as distance from Kumasi increased, consistent with the distance to market hypothesis, prediction 4a. Rodent trade volumes were independent of distance, whereas the rodent:ungulate ratio increased as distance to Kumasi increased (Fig. 2g, Table 3). Over the full survey period, there was a substantial expansion of the market catchment area (Fig. 3a), consistent with prediction 4b: the mean distance from source to market increased by 20% from 37.3 to 44.8 km (*t* = 2.26, df = 314, *p* = 0.025), and the mean distance traveled per carcass increased by 54% from 35.7 to 55.1 km (*t* = 4.23, df = 314, *p* = 0.001).

**Table 3:** **Effect sizes** a **in a generalized linear model, with quasi‐poisson errors, relating landscape characteristics to commercial bushmeat trade volumes**.^b^

Explanatory variable	All species (SE)	Ungulates (SE)	Rodents (SE)	Rodent: ungulate ratio
Intercept^c^	−15.9**	−13.5**	−22***	−3.6***
	(4.8)	(4.7)	(6.1)	(0.2)
Disturbance (H1)	54.0**	43.6**	68.5**	
	(17.0)	(16.4)	(21.5)	
Disturbance^2^ (H1)	−46.0**	−36.7*	−59.0**	
	(14.8)	(14.4)	(18.8)	
Source overlap (H2)	0.2*	0.2**	0.4***	
	(0.09)	(0.09)	(0.09)	
Protected area (H3)^d^				
Distance (H4)	−9.6 × 10^−3^ *	−9.1 × 10^−3^ *		5.0 × 10^−3^ *
	(4.8 × 10^−3^)	(4.9 × 10^−3^)		(2.3 × 10^−3^)
Year (2002)	1.8***	1.4***	2.6***	1.5***
	(0.4)	(0.4)	(0.5)	(0.2)
Year (2002): hunting pressure	−0.52***	−0.51***	−0.53***	
	(0.1)	(0.1)	(0.1)	

a Effect size shows how large the influence of the response variable is and is the coefficient of a variable in the regression. It can be used to compare the relative influence of different response variables.

b Sample size is 338 (all species, ungulates, rodents) and 210 (rodent:ungulate ratio). Bushmeat trade is described in terms of total number of carcasses. Significance: *5%, **1%, ***0.1%.

c Base year 1986 for reference.

d There was no significant effect of protected area for any species group; thus, this row is blank.

## Discussion

Our findings are broadly in line with our hypotheses (Table 1) in 3 out of 4 cases. In the first case, our results highlight the important role disturbed landscapes play in supporting the commercial bushmeat trade around Kumasi; lower trade volumes originated from undisturbed and more heavily disturbed sites relative to semidisturbed sites. By controlling for distance and including a proxy for hunting pressure (source overlap) in our model, this pattern is unlikely to be confounded with either remoteness of market or the relative intensity of hunting. Direct observations of hunting off‐take in other landscapes have reported similar patterns (Wilkie 1989; Demmer & Overman 2001).

In the second case, trade volumes were positively correlated with source overlap. Over time, however, the relationship between source overlap and trade volumes reversed itself substantially, such that later in the study period trade volumes became negatively correlated with source overlap. This result suggests hunting in the study region has had a dramatic effect on productivity, with formerly productive areas now producing little and production from formerly less heavily hunted areas increasing in compensation. Changing patterns of production were also evident in the rodent:ungulate ratio, which increased strongly over time across the landscape. The ratio did not increase disproportionately in more heavily hunted areas as predicted, but taken together these results are broadly consistent with increasing depletion of wildlife across the landscape.

In the third case, the lack of evidence linking protected areas with commercial trade volumes is contrary to our expectations. Although our findings might suggest that the protected areas are safe from exploitation, it is well known that Ghana's forest reserves have been subject to heavy hunting pressure for many years and exhibit very low numbers of large mammals as a result (Struhsaker & Oates 1995; Holbech 2001; Brashares 2003; Gatti 2010). Consequently, the lack of effect of protected areas we found, most likely reflects the fact that previous exploitation now prevents such areas from contributing in any detectable way to the commercial bushmeat trade in Kumasi.

In the fourth case, trade in all species groups except rodents declined with distance from Kumasi and the market catchment expanded significantly over time. The trade in rodents was biased toward more distant locations (contrary to the trade in ungulates). It seems unlikely that the reason for these differences would be that ungulate populations are particularly depleted at the outer edges of the catchment area, particularly because the majority of ungulates traded at Atwemonom are generalist species able to persist in a variety of degraded landscapes. Rather, a more plausible explanation involves trade filters (Allebone‐Webb et al. 2011). In Ghana cane rat is generally the most valuable bushmeat per kilo on the Atwemonom market, in line with consumer preferences (Falconer 1992; Asibey 2000; McNamara 2014). Intermediaries, who deal in bulk and travel long distances, may be more likely to maximize profits by trading primarily in cane rats. Indeed, a previous study of the Atwemonom market described cane rats as being delivered in carloads (Hofmann et al. 1999). Such trading behavior, whereby trade from farther away is biased toward species that are more valuable on a per‐kilo basis (such as cane rats), may explain the positive correlation between distance and the rodent:ungulate ratio. Despite the strong correlation between distance and our proxy for hunting pressure, both predictor variables were significant and opposite in their effect on bushmeat trade volume, in line with expectations. It would therefore appear that their effects were captured appropriately in the model.

The growth in the size of the catchment area suggests hunters are participating in the market from farther afield. This may be due to hunters who previously sold their catch locally now entering the urban market in response to high prices or lower transport costs (Brashares et al. 2001; Kramer et al. 2009). It may also be due to wildlife depletion, together with better job opportunities, reducing the incentive to hunt closer to the city. Some of the villages that supplied large amounts of bushmeat to Kumasi in the early years are now effectively suburbs of the city, with local residents pursuing urban employment. First hand information on what has driven the growth in catchment area would contribute to a more mechanistic understanding of the dynamics of this system.

There was a small but significant increase in trade across all species groups between 1986 and 2002. However declines in trade from heavily hunted areas, changes in species composition, increasing dominance of nonforest species in the market data set, in particular the cane rat (Supporting Information), and a growing catchment area point to an evolution of the system to an unfavorable state for both wildlife and people that might not be immediately evident from a straightforward assessment of trade biomass. We examined how the number of animals traded varied across the landscape rather than how biomass varied. Thus, the growth in trade for all species may be due in part to the growing influence of smaller, more numerous rodent species, notably the cane rat. Breaking down trade data into taxonomic groups helps isolate such effects, but to fully assess changes in overall productivity of the system, biomass would be an interesting additional metric.

Our results highlight the increasingly important role semidisturbed landscapes can play in supporting the bushmeat trade and the increasingly dominant role of rodent species (particularly the cane rat) in the market. Socio‐economically this is important. The bushmeat trade in Ghana is a multi‐million dollar industry (Ntiamoa‐Baidu 1998) and continues to contribute substantially to livelihoods. Our findings emphasize the need to consider bushmeat production in the farm‐bush matrix when assessing ecosystem service priorities and undertaking conservation planning exercises (e.g., Holbech 2001; Vandermeer & Perfecto 2007; Fischer et al. 2008).

If bushmeat production is to be appropriately incorporated into land‐use policies, more information is needed to understand its value and production potential in the agricultural matrix and the degree to which current levels of exploitation are sustainable in the face of the current population increase in Ghana and the region at large. If realized effectively such policies should lead to the promotion of beneficial ecosystem services and the enhancement of both ecological and livelihood resilience. The development of policies that acknowledge and promote the inherent biodiversity value of anthropogenic tropical forest systems is crucial, not just for Ghana, but for many developing countries in the tropics, where human‐modified landscapes are increasingly likely to be the future face of many rural regions and where the need to balance the objectives of conservation and development will be of great importance (McNeely & Scherr 2003; Balmford et al. 2005; Norris et al. 2010).

## Supporting information

A description of market survey methods (Appendix S1), summary of market data for 1986 and 2002 (Appendix S2), detailed description of the image processing and classification methodology (Appendix S3), summary of model variables (Appendix S4), and the correlation matrix describing relationships between model variables (Appendix S5) are available online. The authors are solely responsible for the content and functionality of these materials. Queries (other than absence of the material) should be directed to the corresponding author.
