# Supplementary material for: Long‐term spatio‐temporal changes in a West African bushmeat trade system
Source: Conserv Biol. 2015 Jun 23;29(5):1446–57. doi: 10.1111/cobi.12545 (PMC4745032; doi:10.1111/cobi.12545)
Supplement: Supplementary file 1 — A description of market survey methods (Appendix S1), summary of market data for 1986 and 2002 (Appendix S2), detailed description of the image processing and classification methodology (Appendix S3), summary of model variables (Appendix S4), and the correlation matrix describing relationships between model variables (Appendix S5) are available online. The authors are solely responsible for the content and functionality of these materials. Queries (other than absence of the material) should be directed to the corresponding author. [file COBI-29-1446-s001.pdf]

Supplementary Materials

Appendix S1 - Description of market data collection

Appendix S2 – Summary of 1978 – 2004 market data

Appendix S3 – Satellite image preparation and processing

Appendix S4 – Model variable summary statistics

Appendix S5 – Correlation matrix of model variables

References

Appendix S1

Description of market data collection

Description of the dataset

Between 1978 and 2004, officials from the Ghana Wildlife Division (GWD), in particular paper co-author Acheampong Brenyah (AB) regularly surveyed the daily trade passing through Atwemonom. Data were collected as hunters arrived at the market. The market has a central preparation area, where, following the transaction between hunter and market woman, the bushmeat is prepared and divided up to be returned to the market women for sale (Falconer 1992). This central processing system allowed observers to monitor efficiently the trade passing through the market. Information was recorded on the species, carcass weight, wholesale price received by the hunter and location from where the hunter had come. The dataset therefore represents a spatially explicit record of the commercial trade passing through the market over a 26-year period. The full dataset represents 86,365 records made over 4,965 days and 268 months, covering 26 years from May 1978 to June 2004.

Caveats

There are a number of important caveats covering the data that need to be considered for analysis. The first of these arises due to the presence of the annual close season. During this time, only the cane rat (known locally as grasscutter) can be legally traded. Although other species are sporadically recorded in the data during this period, discussion with members of the team who monitored the market indicated that the recording of banned species (i.e. illegal trade) was unlikely to be reliable. In short, as the monitoring team required the trust of the market women to operate, there was an incentive to

ignore elements of the illegal trade. For this reason, data from the close season was excluded from the following analysis. The resulting dataset, covering only the open season, consisted of 67,438 records, over 3,335 days and 180 months. Table 2.2 summarises the species break down of the data.

A second caveat applies to the species records. Just as observers turned a blind eye to illegal trade during the closed season, so they also reported turning a blind eye to trade in schedule 1 species, which are fully protected by law at all times of the year. During personal observation of the market by JMcN and AB in 2011, a number of schedule 1 species were openly traded, including pangolin species, *Manidae spp.,* and the African civet, *Civettictis civetta.* Neither of these species is common in the market data recorded between 1978 and 2004. Indeed, there is only one recorded pangolin transaction in 26 years, yet personal observations over a number of days saw pangolins being traded daily. It is therefore unlikely that the market data represents a true record of the trade in schedule 1 species.

The resultant database, is strongly skewed towards seven commonly traded species, which constitute 94% of the trade by volume, namely the cane rat or grasscutter, Maxwell's duiker, royal antelope, bushbuck, black duiker, brush-tailed porcupine and giant pouched rat.

Observer Effort

When visited, the market was reported to have been monitored from the time it opened until it closed. However, the distribution of observation days throughout the study period is not consistent (figure 2.3). Observation effort in the latter half of data, from 1995 onwards, is notably lower, with only 16 days monitored in

1997. In the eight months relating to each of the open seasons of years included in the spatial model, the number of days observed were: 1986: 176; 1987: 183; 2002: 119; 2003: 89.

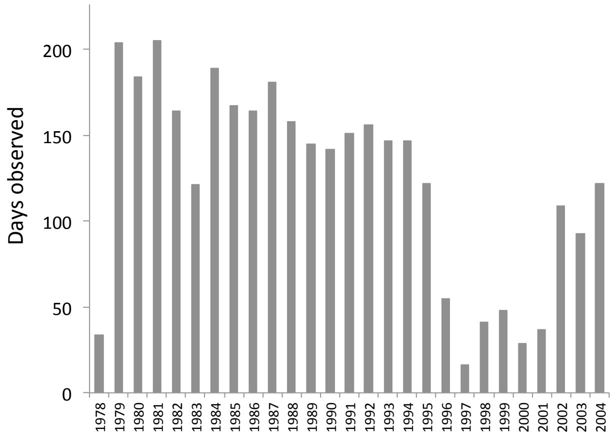

Figure S1 Number of days during the open season on which Wildlife Division staff visited the market

One-off survey

During the one-off survey in 2011, the same method was used as in the long-term dataset, as confirmed by AB, who participated in both data-collection exercises. Trade passing through the Atwemonom market was surveyed during a one-week period in June 2011. The market was surveyed from opening at 5am until closing at 4pm. Data were gathered on wholesale price, weight, species, and the location from which the hunter travelled. Monitoring of the market is relatively straightforward as hunters bring carcasses to market whole and negotiate the sale at a small central processing area, which provides a focal point at which observers can record the details of the transaction and details of capture.

Appendix S2

Summary of 1978 – 2004 market data

Table S1: Summary of data of records from the Atwemonom Market, Kumasi during the open season (December – July inclusive), during the 1986 and 2002 periods. Species are sorted according to the number of records in 2002. For a breakdown of species traded over the entire period 1978 to 2004, please see (McNamara 2014). Nomenclature for animal names taken from (Kingdon 2003).

**All Species**	**Latin Name**	**1986Records**	**2002 Records**
Cane rat	*Thryonomys swinderianus*	693	1314
Bushbuck	*Tragelaphus scriptus*	534	421
Maxwell duiker	*Cephalophus maxwelli*	843	401
Royal antelope	*Neotragus pygmaeus*	839	159
Brush-tailed porcupine	*Atherurus africanus*	254	149
Black duiker	*Cephalophus niger*	484	122
Giant pouched rat	*Cricetomys spp.*	307	114
Red-flanked duiker	*Cephalophus rufilatus*	4	25
Long-nosed mongoose	*Herpestes naso*	13	14
Bay duiker	*Cephalophus dorsalis*	0	12
Mona monkey	*Cercopithecus mona*	57	12
Red river hog	*Potamochoerus porcus*	1	10
Spot-nosed monkey	*Cercopithecus petaurista*	9	9
Ground squirrel	*Euxerus erythropus*	31	7
Pel's anomalure	Anomalurus peli	13	4
Common duiker	*Cephalophus sylvicapra*	0	2
Kob	*Kobus kob*	0	1
Marsh mongoose	*Atilax paludinosus*	6	1
Fruit bat spp	*Pteropodidae spp*	0	1
African civet	*Civettictis civetta*	69	0
Slender mongoose	*Herpestes sanguineus*	4	0
Forest genet	*Genetta maculata.*	67	0
Francolin	*Francolinus* spp.	2	0
Palm civet	*Nandinia binotata*	102	0
**Rodent Species**			
Cane rat	*Thryonomys swinderianus*	693	1314
Brush-tailed porcupine	*Atherurus africanus*	254	149
Giant pouched rat	*Cricetomys spp.*	307	114
Ground squirrel	*Euxerus erythropus*	31	7
Pel's anomalure	Anomalurus peli	13	4
**Ungulates**			
Long-nosed mongoose	*Herpestes naso*	13	14
Mona monkey	*Cercopithecus mona*	57	12
Red river hog	*Potamochoerus porcus*	1	10
Spot-nosed monkey	*Cercopithecus petaurista*	9	9
Marsh mongoose	*Atilax paludinosus*	6	1
Fruit bat spp	*Pteropodidae spp*	0	1
African civet	*Civettictis civetta*	69	0
Slender mongoose	*Herpestes sanguineus*	4	0
Rusty-spotted genet	*Genetta maculata*	67	0
Francolin	*Francolinus* spp.	2	0
African palm civet	*Nandinia binotata*	102	0

Appendix S3

Satellite image preparation and processing

Semi-processed, georeferenced Landsat satellite images were obtained online from the USGS Global Visualisation Viewer (Glovis) for 1986 and 2002. Landsat imagery is recognised as an effective basis for analysing patterns of land-use and land change (Tucker & Townshend 2000). The Kumasi catchment area, as defined by the bushmeat market data, covered an area of 39,204km^2 (198km x 198km) and intersected 4 Landsat scenes (table S1).

Table S2: Attributes of Landsat images

Year	Image	Date of Acquisition	Row	Path	Resolution	Level of Processing
1986	Landsat MSS	December 1986	055 & 056	194	60m	L1T
	Landsat MSS	December 1986	055 & 056	195	60m	L1T
2002	Landsat TM+	January 2002	056	194	30m	L1T
	Landsat TM+	December 2002	055 & 056	195	30m	L1T
	Landsat TM+	February 2003	055	194	30m	L1T

The range of dates for the 2002 image composite was necessary to find high-quality, cloud-free images. These images represented the most closely related combination in terms of season, dates and scanner for the study site in our time frame. Data for the 1986 period was from the Landsat Multispectral Scanner (MSS), and for 2002, the Landsat Thematic Mapper scanner (TM).

Image preparation

Images were prepared in IDRISI. For 1986, three bands were selected to produce false colour composite images, band 1 (green) band 2 (blue) and band 4 (red), in line with the convention for the analysis of vegetation using Landsat MSS images (De Fries et al. 1998). For 2002, Bands 2 (blue), band 3 (green) and band 4 (red) were selected, in line with convention for the analysis of vegetation using Landsat TM images (Yiran et al. 2012). A single false colour composite image of

the study area was produced for each time period and analysed by eye for consistency. Due to either differences in dates when scenes were taken, or haze, a single classification procedure (based on the merging of individual scenes into a single mosaic prior to image clustering and cluster labelling), was deemed inappropriate due to spectral differences between scenes. An unsupervised classification, whereby land classes are separated through the use of an automated algorithm that analyses the spectral bands, highlighted this inconsistency. Thus scenes were cropped into sub-scenes, which were classified separately prior to composing the final image, based in part on the methodology implemented in Guindon & Edmonds (2002). For classification purposes, 1986 was divided into three sub-scenes, and 2002 into four sub-scenes.

Pre-classification of land cover

Prior to classification, the spectral bands produced by the MSS scanner for the 1986 time period were assessed for signs of noise. Bands 1 and 2 exhibited noise in all scenes. Band 4 was free from sensor error. A Principal Component Analysis (PCA) was performed within IDRISI to reduce this error. The analysis showed that in all scenes the first component accounted for the vast majority of the variation (scene 1, 98.2%, scene 2, 97.3%, scene 3, 97.8%). Comparison of image quality with the inclusion of the second component showed a noticeable increase in noise. Thus, of the three computed components, only the first was selected for the reproduction of bands 1 and 2.

Classification of land cover

Supervised classification techniques were adopted based on their suitability for quantitative analysis of remotely sensed images (Lillesand et al. 2004). A

ground-truthing exercise was conducted in one part of the study site. GPS markers were recorded for different land-use classes and photographs taken of the surrounding vegetation for later review purposes. 21 markers were recorded; 4 in closed canopy forest, 6 in areas of open canopy forest and tree crops, 3 within settlements and areas of bare earth, 8 within farmland (both fallow and productive). This ground-truthing exercise was augmented by additional assessment methods including the use of the Google Earth application and consultation with experts familiar with the study site at the Department of Geography, University of Ghana (Kusimi 2008).

Eight classes were initially defined: Closed canopy forest, open canopy forest and tree crops, settlements and bare earth, fallow farmland, productive and recently harvested farmland, savannah and water. Analysis of the signatures for different land classes using the graphical functions SIGCOMP and SCATTER showed the distinction between farm classes to be poor. Thus farmland was grouped into a single classification for the final analysis. The inability to distinguish farm classes reliably is not surprising when one considers the scale of many farm plots in Ghana (a few hectares), and the resolution of the scanners 60m x 60m for MSS (30m x 30m for TM). Cloud, which was present in a small section of one scene in 2002 (representing < 6.4% of the image area), was classified as "No Data".

The separability of the remaining seven digitised classes was quantified using the Jeffries-Matusita Distance (or the Transformed Divergence Measures, TDM) of signature separability. These measures quantify the degree of overlap between signatures in a pairwise fashion on a scale from 0 to 2 (0 – 2000 for the TDM) with 2 (or 2000) being complete separation. Values greater than 1.9 (or

1900) indicate good separability, and between 1 and 1.9 (or 1000 and 1900) moderate separability. Signature separability was satisfactory for all classified classes, falling between 1.8 and 1.98 (or 1870 and 1959).

Scenes were classified using a maximum likelihood modelling routine. Following classification, individual scenes were composited into a single unified image. Due to differences in image resolution of the MSS and TM scanners, the 1986 image was resampled to a 30m x 30m pixel resolution in line with the native resolution of the TM scanner associated with the 2002 image. The total Root Mean Square (RMS) error describing the probability that the control points used in the resampling process vary from their true position was within acceptable limits, (RMS = 0.001, limit of acceptability = 0.5).

Appendix S4

Model variable summary statistics

Table S3: Summary of model variables extracted from the data, *V* is the response variable and *d*, *r*, *h*, *l* the independent variables

Symbol	Data	Mean	Median	SD	Range	Units
V	Bushmeat volume.					
	All Species	11.3	3.7	18.1	0.5-88.3	Carcass/day
	Ungulates	6.5	2.8	9.8	0.5-81.4	Carcass/day
	Rodents	6.9	2.8	10.9	0.5-13.4	Carcass/day
	Rodent:Ungulate ratio	1.3	0.8	1.8	0.05-11	none
d	Habitat disturbance index	0.6	0.6	0.08	0.4-0.9	none
h	Source overlap	3.2	3.2	1.6	1-6.8	km^2
r	Protected area coverage	0.07	0.0	0.1	0-0.7	km^2
l	Distance to market	47.5	40.1	30.4	6.2-172.5	km

Appendix S5

Correlation matrix of model variables

Table 4: Correlation matrix of model variables. Values are Pearson correlation coefficients

	Disturbance	Distance	Source overlap	Protected Area	Year
Disturbance	1	**0.33** [a]	**-0.38**[b]	**0.47**[c]	-0.29
Distance		1	**-0.79**[d]	**0.42**[e]	-0.04
Source overlap			1	**-0.48**	-0.00
Protected Area				1	-0.05

[a] $p < 0.01$, $t = 6.47$, d.f = 337
[b] $p < 0.01$, $t = -7.32$, d.f = 337
[c] $p < 0.01$, $t = 9.89$, d.f = 337
[d] $p < 0.01$, $t = -0.69$, d.f = 341
[e] $p < 0.01$, $t = 8.89$, d.f = 341
[f] $p < 0.01$, $t = -10.56$, d.f = 341

Falconer, J. (1992) Non-timber forest products in southern Ghana. Natural Resources Institute, Kent, UK.

De Fries, R.S., Hansen, M., Townshend, J.R.G. & Sohlberg, R. (1998) Global land cover classifications at 8 km spatial resolution: The use of training data derived from Landsat imagery in decision tree classifiers. *International Journal of Remote Sensing*, **19**, 3141–3168.

Guindon, B. & Edmonds, C. (2002) Land-rea land-cover mapping through scene based classification compositing. *Photogrammetric Engineering & Remote Sensing*, **68**, 589 – 596.

Kingdon, J (2003) The Kingdon Field Guide to African Mammal*s*, Helm, pp476

Kusimi, J. (2008) Assessing land use and land cover change in the Wassa West District of Ghana using remote sensing. *GeoJournal*, **71**, 249–259.

Lillesand, T.M., Kiefer, R.W. & Chipman, J.W. (2004) *Remote Sensing and Image Interpretation*. Wiley.

McNamara, J. (2014) The dynamics of a bushmeat hunting system under social, economic, and environmental change. *Unpublished PhD thesis, Imperial College London.*

Tucker, C.J. & Townshend, J.R.G. (2000) Strategies for monitoring tropical deforestation using satellite data. *International Journal of Remote Sensing*, **21**, 1461–1471.

Yiran, G.A.B., Kusimi, J.M. & Kufogbe, S.K. (2012) A synthesis of remote sensing and local knowledge approaches in land degradation assessment in the Bawku East District, Ghana. *International Journal of Applied Earth Observation and Geoinformation*, **14**, 204–213.
